# Aberrant Cerebellar Circuitry in the Spinocerebellar Ataxias

**DOI:** 10.3389/fnins.2020.00707

**Published:** 2020-07-16

**Authors:** Katherine J. Robinson, Maxinne Watchon, Angela S. Laird

**Affiliations:** Centre for Motor Neuron Disease Research, Department of Biomedical Science, Faculty of Medicine, Health and Human Sciences, Macquarie University, Sydney, NSW, Australia

**Keywords:** spinocerebellar ataxia, neurodegeneration, Purkinje cell vulnerability, Purkinje cell dysfunction, cerebellar circuitry, disease mechanisms, cerebellar pathophysiology

## Abstract

The spinocerebellar ataxias (SCAs) are a heterogeneous group of neurodegenerative diseases that share convergent disease features. A common symptom of these diseases is development of ataxia, involving impaired balance and motor coordination, usually stemming from cerebellar dysfunction and neurodegeneration. For most spinocerebellar ataxias, pathology can be attributed to an underlying gene mutation and the impaired function of the encoded protein through loss or gain-of-function effects. Strikingly, despite vast heterogeneity in the structure and function of disease-causing genes across the SCAs and the cellular processes affected, the downstream effects have considerable overlap, including alterations in cerebellar circuitry. Interestingly, aberrant function and degeneration of Purkinje cells, the major output neuronal population present within the cerebellum, precedes abnormalities in other neuronal populations within many SCAs, suggesting that Purkinje cells have increased vulnerability to cellular perturbations. Factors that are known to contribute to perturbed Purkinje cell function in spinocerebellar ataxias include altered gene expression resulting in altered expression or functionality of proteins and channels that modulate membrane potential, downstream impairments in intracellular calcium homeostasis and changes in glutamatergic input received from synapsing climbing or parallel fibers. This review will explore this enhanced vulnerability and the aberrant cerebellar circuitry linked with it in many forms of SCA. It is critical to understand why Purkinje cells are vulnerable to such insults and what overlapping pathogenic mechanisms are occurring across multiple SCAs, despite different underlying genetic mutations. Enhanced understanding of disease mechanisms will facilitate the development of treatments to prevent or slow progression of the underlying neurodegenerative processes, cerebellar atrophy and ataxic symptoms.

## Introduction

The spinocerebellar ataxias (SCAs) are a diverse group of neurodegenerative diseases that share clinical phenotypes including impaired balance and motor coordination, Purkinje cell death and cerebellar atrophy (Hekman and Gomez, 2015; Prestori et al., 2019). The global prevalence of SCA is estimated at three affected patients per 100 000 individuals (Ruano et al., 2014), with higher prevalence in some geographical regions (Hekman and Gomez, 2015; Sullivan et al., 2019).

For most cerebellar ataxias, pathology can be attributed to an underlying genetic mutation. These known genetic causes include glutamine-encoding CAG repeat expansions (polyQ), untranslated repeat expansions, DNA rearrangements and conventional mutations, such as missense mutations, insertions or deletions (Hekman and Gomez, 2015; Paulson et al., 2017). As with many other inherited disorders, there are several different cellular processes and pathways that contribute to disease pathogenesis. Such processes and pathways include transcriptional dysregulation, RNA toxicity, proteostasis, protein aggregation and altered neuronal activity (Hekman and Gomez, 2015; Paulson et al., 2017). Despite vast heterogeneity in causative genes across the different SCAs, there is considerable overlap in the cellular processes affected, producing similar neuropathology and clinical phenotypes. Nevertheless, our understanding of the contribution of the various pathogenic pathways or mechanisms to each SCA disease remains limited.

Dysfunction of Purkinje cells and consequent dysfunction of cerebellar circuitry is a common feature of the SCAs, regardless of the disease-causing gene mutation (Kasumu and Bezprozvanny, 2012; Hekman and Gomez, 2015). Despite ubiquitous expression of most SCA-causing genes throughout the brain, pathology is most prominent within the cerebellum (Bettencourt et al., 2014). This suggests that neuronal populations within the cerebellum, particularly Purkinje cells, may be more susceptible to changes in transcription, translation, protein quality control and signaling than other neuronal cell types. Furthermore, Purkinje cell degeneration has been found to precede other important disease phenotypes, including movement and balance deficits. This review will discuss why the cerebellum may be particularly vulnerable or susceptible to the cellular and mechanistic insults caused by SCA-causing disease mutations.

## Clinical Classification of the Spinocerebellar Ataxias

Currently, there are over forty-five distinct types of SCA that have been clinically described (Table 1). The most common clinical symptoms associated with SCA include gait ataxia and uncoordinated movements (Sullivan et al., 2019). In most SCA patients, gait ataxia is the first sign of dysfunction. Other symptoms often associated with the different SCAs include dysarthria, nystagmus, vision impairments, pyramidal and extrapyramidal signs, ophthalmoplegia and cognitive deficits (Sullivan et al., 2019). Despite the commonality of the presence of ataxia, there is nevertheless considerable variability in disease symptoms across the SCA diseases, leading these diseases to be clinically classified into subtypes; autosomal dominant cerebellar ataxia type I, II and III.

**TABLE 1:** Summary of each identified form of Spinocerebellar ataxia, including causative gene, type of genetic mutation, effect of genetic mutation, clinical symptoms and key references.

SCA	Gene	Mutation	Effect of mutation	Impaired/Altered processes	Clinical symptoms	Clinical classification	Key references
SCA1	*ATXN1*	Trinucleotide repeat expansion (39-83 repeats)	Hypothesized gain-of-function. Downregulation of genes associated with maintenance of calcium homeostasis.	Dysregulation of transcription. Downstream glutamate signaling is indirectly dysregulated, resulting in reduced Purkinje cell firing frequency.	Pyramidal signs, peripheral neuropathy and opthalmoparesis. Loss of > 75% of Purkinje cells.	Type I	Orr et al., 1993; Lin et al., 2000; Lam et al., 2006; Power et al., 2016; Rousseaux et al., 2018
SCA2	*ATXN2*	Trinucleotide repeat expansion (32-79 repeats)	Hypothesized toxic gain-of-function.	Toxic enhancement of mGluR and IP3R1 function, increasing calcium release and Purkinje cell hyperexcitability.	Hyporeflexia, dysarthria, tremor and slow eye movements. Loss of > 75% of Purkinje cells.	Type I	Imbert et al., 1996; Pulst et al., 1996; Sanpei et al., 1996; Liu et al., 2009; Meera et al., 2017
SCA3	*ATXN3*	Trinucleotide repeat expansion (> 40 repeats)	Hypothesized loss of deubiquitinase activity. Gain of IP3R1 function.	Longer ubiquitin chains and dysregulation of transcription. Mutant ataxin-3 interacts with IP3R1, causing increased calcium release and Purkinje cell excitability.	Progressive gait imbalance, speech difficulties, ocular motor difficulties, spasticity, dystonia, dysarthria and dysphagia. Loss of ∼25% of Purkinje cells.	Type I	Kawaguchi et al., 1994; Paulson et al., 1997; Chen et al., 2008
SCA4	Chromosome 16	Unknown	*NHE5* is a hypothesized candidate gene.	Hypothesized to disrupt Na^+^/H^+^ exchange in skeletal muscles, leading to altered intracellular pH and cell death.	Sensory peripheral neuropathy, extensor plantar responses, areflexia, dysarthria.	Type I	Flanigan et al., 1996; Higgins et al., 1997
SCA5	*SPTBN2*	Missense mutation	Dominant negative effects on sodium channel complexes. Impaired mGluR1 and EAAT4 expression.	Impaired long-term potentiation of Purkinje cells. Decreased responsiveness to mGluR1 and excessive glutamate presence. inducing toxicity. Abnormal Purkinje cell dendritic development.	Pure cerebellar ataxia, with incoordination of extremities and slurred speech. Relatively mild severity.	Type III	Ranum et al., 1994; Ikeda et al., 2006; Perkins et al., 2010; Armbrust et al., 2014
SCA6	*CACNA1A*	Trinucleotide repeat expansion (19-33 repeats)	Toxic loss of α1ACT function, a transcription factor.	Reduced expression of genes critical to Purkinje cell development and survival.	Pure cerebellar ataxia. Loss of > 75% of Purkinje neurons.	Type III	Zhuchenko et al., 1997; Matsuyama et al., 1999; Toru et al., 2000; Du et al., 2013; Du et al., 2019
SCA7	*ATXN7*	Trinucleotide repeat expansion (38-150 repeats).	Loss of chromatin remodeling and Bergmann glial cell function.	Impaired regulation of transcription and glutamate-reuptake, resulting in dysfunction of Purkinje cells and retinal cells.	Dystrophy of retinal rods and cones, resulting in vision loss, hypoacusia and hypotonia.	Type II	David et al., 1997; Benton et al., 1998; Helmlinger et al., 2004; Matilla-Dueñas et al., 2014
SCA8	*ATXN8*	Non-coding trinucleotide repeat expansion (107-127 repeats)	Toxic gain-of-function (RNA and protein products).	Repeat-associated non-ATG (RAN) translation. Formation of ribonuclear inclusions which localize to RNA binding protein Mbn11.	Cognitive dysfunction, myotonic dystrophy, pyramidal and sensory signs.	Type I	Koob et al., 1999
SCA10	*ATXN10*	Non-coding pentanucleotide repeat (800-4500 repeats)	Gain of toxic RNA function. Loss of hnRNPK function (RNA splicing factor).	Neuritogensis, accumulation of aggregated RNA and increased activation of apoptotic cascades.	Ataxic gait, dysarthria, nystagmus, hypotonia and occasional epilepsy.	Type I	Matsuura et al., 1999; Zu et al., 1999; Matsuura et al., 2000
SCA11	*TTBK2*	Frameshift mutation	Loss of TTBK2 function, resulting in reduced TTBK2 transcript levels.	Mutation causes loss of function, impacting on tau regulation/phosphorylation and neuronal integrity.	Pure cerebellar ataxia, pyramidal signs	Type III	Houlden et al., 2007
SCA12	*PPP2R2B*	Non-coding trinucleotide repeat expansion (55-78 repeats)	Unknown	Yet to be clarified, however hypothesized mitochondrial dysfunction and increased oxidative stress.	Gait ataxia, upper limb postural tremor, hyperreflexia, parkinsonian features and dementia.	Type I	Holmes et al., 1999; O’Hearn et al., 2001
SCA13	*KCNC3*	Missense mutation	Both loss and gain of Kv3.3 function.	Activation curve of Kv3.3 shifted toward channel opening and slowed channel closing, disrupting Purkinje cell excitability and function.	Cerebellar ataxia, dysarthria, nystagmus, pyramidal signs, delayed motor and cognitive development.	Type I	Herman-Bert et al., 2000; Waters et al., 2006; Figueroa et al., 2010; Irie et al., 2014
SCA14	*PRKCG*	Missense mutation	Both loss and gain of PKCγ function.	Altered activity PKCγ, resulting in altered extracellular Ca^2+^ entry through TRPC3 receptors.	Cerebellar ataxia, myoclonus and task-specific dystonia.	Type I	Chen et al., 2003; Verbeek et al., 2005; Adachi et al., 2008
SCA15/16	*ITPR1*	Missense mutation	Loss of IP3R1 function.	Reduced intracellular calcium signaling, decreasing Purkinje cell excitability.	Pure cerebellar ataxia, tremor and cognitive impairment	Type III	Storey et al., 2001; van de Leemput et al., 2007; Iwaki et al., 2008
SCA17	*TBP*	Trinucleotide repeat expansion (47-63 repeats)	Altered TATA-box binding protein function.	Impaired transcription. Effect on Purkinje cells is still an area of active research.	Gait ataxia, dysmetria, hyperreflexia, parkinsonian features and dementia.	Type I	Nakamura et al., 2001
SCA18	*IFRD1*	Missense mutation	Hypothesized loss of function.	Abnormalities in IFRD1 protein folding, increased nuclear localization and hypothesized impairments to normal transcriptional co-repressor function.	Gait ataxia, sensory neuropathy, dysmetria, dysarthria and nystagmus.	Type I	Brkanac et al., 2002; Brkanac et al., 2009; Lin et al., 2018
SCA19/22	*KCND3*	Missense mutation	Loss of Kv4.3 function.	Kv4.3 mislocalization, resulting in lack of expression at PC synapse, impairing long term potentiation	Cerebellar ataxia, hyporeflexia and myoclonic movements.	Type I	Verbeek et al., 2002; Chung et al., 2003; Duarri et al., 2012; Lee et al., 2012
SCA20	Chromosome 11	Genomic duplication	Hypothesized gain of *DAGLA* function.	*DAGLA* is expressed in Purkinje cells and acts to weaken glutamate signaling.	Cerebellar ataxia, dysarthria and spasmodic dysphonia.	Type I	Knight et al., 2004
SCA21	*TMEM240*	Missense mutation and/or stop mutation	Hypothesized gain of function.	Synaptic transmembrane protein, function currently unknown.	Slow progressing ataxia, mild to severe cognitive impairment and parkinsonian features.	Type I	Devos et al., 2001; Vuillaume et al., 2002; Delplanque et al., 2014
SCA23	*PDYN*	Missense mutation	Increase in DynA production, toxic gain-of-function.	Increased opioid and glutamate signaling, producing downstream glutamate toxicity.	Gait ataxia, dysarthria, dysmetria and hyperreflexia.	Type I	Verbeek et al., 2004; Bakalkin et al., 2010
SCA25	Chromosome 2	Unknown	Unknown	Candidate gene: *CRIPT2*. Encoded protein is involved in postsynaptic architecture.	Cerebellar ataxia, peripheral neuropathy.	Type I	Stevanin et al., 2005
SCA26	*EEF2*	Missense mutation	Increased rate of protein frameshift during translation.	Structural defect in eEF2 protein, resulting in impaired translation and reduced proteostatic capacity.	Pure cerebellar ataxia and dysarthria. Loss of > 75% of Purkinje cells.	Type III	Yu et al., 2005; Hekman et al., 2012
SCA27	*FGF14*	Missense mutation	Loss of Nav1.6 expression, dominant negative effect on Cav2.1 and Cav2.2.	Reduced expression of ion channels, leading to reduced ion influx and suppression of Purkinje cell output.	Early onset upper limb postural tremor, dyskinesia, dystonia and slowly progressing CA.	Type I	van Swieten et al., 2003; Laezza et al., 2007; Shakkottai et al., 2009; Yan et al., 2013
SCA28	*AFG3L2*	Missense mutation	Dominant-negative and loss-of-function effects.	Loss of mitochondrial ATP-dependent protease activity, resulting in reduced degradation of misfolded proteins within the mitochondria and impaired mitochondrial function.	Ataxic gait, nystagmus, ptosis and opthalmoparesis.	Type I	Cagnoli et al., 2006; Mariotti et al., 2008; Cagnoli et al., 2010
SCA29	*ITPR1*	Missense mutation	Dominant negative effect on IP3R1.	Downregulation of IP3R1, and decreased calcium release. One mutation found to enhance calcium release.	Gait ataxia, dysarthria, ophthalmoplegia, pyramidal and extrapyramidal dysfunction and cognitive impairments.	Type I	Huang et al., 2012; Ando et al., 2018
SCA30	Chromosome 4	Unknown			Slow progressing, pure cerebellar ataxia with minor pyramidal signs.	Type III	Storey et al., 2009
SCA31	Chromosome 16	Intronic pentanucleotide repeat expansion	Hypothesized RNA-mediated toxic gain-of-function.	Repeat-expanded RNA found to localize with centromeres. Unknown effect on Purkinje cells.	Pure cerebellar ataxia, relatively late onset (average age: 61.2 years).	Type III	Ouyang et al., 2006; Sato et al., 2009
SCA34	*ELOVL4*	Missense mutation	Unknown	Impaired differentiation of skin cells. Hypothesized impacts on proteostasis.	Ataxic gait, nystagmus, dysarthria and erythron-keratodermia.	Type I	Giroux and Barbeau, 1972; Cadieux-Dion et al., 2014; Ozaki et al., 2015
SCA35	*TGM6*	Missense mutation	Loss of transglutaminase 6 enzymatic activity.	Disrupted proteostasis; induction of unfolded protein response, formation of insoluble protein aggregates.	Ataxic gait, mild dysarthria and tremor, dysmetria and hyperreflexia.	Type I	Wang et al., 2010; Tripathy et al., 2017
SCA36	*NOP56*	Intronic hexanucleotide repeat expansion (1500-2000 repeats)	Toxic gain-of-function (RNA toxicity).	RAN translation. Decreased transcription of gene 19bp upstream of expansion, *MIR1292*. Decreased *MIR1292* associated with upregulation of glutamate receptors and perturbed Purkinje cell function.	Cerebellar ataxia with motor neuron involvement, dysarthria and tongue atrophy.	Type I	Kobayashi et al., 2011; Ikeda et al., 2012
SCA37	*DAB1*	Intronic pentanucleotide repeat expansion (31-75 repeats)	Hypothesized RNA-mediated toxicity.	Formation of RNA aggregates. Protein involved in maturation of dendritic spines and synaptogenesis of cerebellar granule cells.	Pure cerebellar ataxia with altered vertical eye movements.	Type III	Serrano-Munuera et al., 2013; Seixas et al., 2017
SCA38	*ELOVL5*	Missense mutation	Hypothesized toxic gain-of function.	Disrupted lipid metabolism, mislocalization of ELOVL5 protein and activation of unfolded protein response.	Ataxic gait, nystagmus, dysarthria and mild sensory neuropathy.	Type I	Di Gregorio et al., 2014
SCA39	Chromosome 11	Chromosomal duplication	Unknown		Cerebellar ataxia, dysmetria, spasticity and dysarthria. Motor impairments from infancy.		Johnson et al., 2015
SCA40	*CCDC88C*	Missense mutation	Hypothesized toxic gain-of-function.	Impaired WNT signaling, JNK hyperphosphorylation, proteolytic cleavage of caspase 3 and increased apoptosis.	Ataxic gait, dysarthria, hyperreflexia, ocular dysmetria and tremor.	Type I	Tsoi et al., 2014
SCA41	*TRPC3*	Missense mutation	Toxic gain of TRPC3 function.	Enhanced permeability of calcium, resulting in increased activation of mGluRs and glutamate induced toxicity.	Pure cerebellar ataxia.	Type III	Fogel et al., 2015
SCA42	*CACNA1G*	Missense mutation	Loss of Cav3.1 function.	Impairments to Purkinje cell rebound bursting, requiring higher current influx to evoke Purkinje cell depolarization.	Ataxic gait, dysarthria nystagmus, pyramidal signs and cognitive impairment.	Type I	Coutelier et al., 2015; Morino et al., 2015; Hashiguchi et al., 2019
SCA43	*MME*	Missense mutation	Hypothesized gain-of-function.	Hypothesized interaction with Schwann cells.	Cerebellar ataxia and peripheral neuropathy.	Type I	Depondt et al., 2016
SCA44	*GRM1*	Missense mutation	Toxic gain of mGluR1 function.	Excessive mGluR1 signaling resulting in increased downstream calcium signaling.	Cerebellar ataxia, corticospinal tract involvement, dysarthria, dysphagia and dysmetria.	Type I	Watson et al., 2017
SCA45	*FAT2*	Multiple missense mutation	Unknown	Hypothesized role in autophagy.	Pure cerebellar ataxia	Type III	Nibbeling et al., 2017
SCA46	*PLD3*	Missense mutation	Hypothesized loss of function.	Hypothesized interaction with β-amyloid.	Cerebellar ataxia and peripheral neuropathy.	Type I	van Dijk et al., 1995; Nibbeling et al., 2017
SCA47	*PUM1*	Missense mutation	Loss of PUM1function.	PUM1 is a RNA binding protein, which interacts with ataxin-1. Reduced expression of *PUM1* results in increased expression of *ATXN1*.	Slow progressing, pure CA with ataxic dysarthria, limb dysmetria and impaired gait.	Type III	Gennarino et al., 2018; Lai et al., 2019
SCA48	*STUB1*	Frame shift mutation	Impaired function of CHIP, an E3 ubiquitin ligase.	Hypothesized dysfunction of protein quality control pathways.	Adult-onset cerebellar ataxia, dystonia, epilepsy, parkinsonism and cognitive impairments.	Type I	Genis et al., 2018; De Michele et al., 2019; Lieto et al., 2020; Palvadeau et al., 2020

Autosomal dominant cerebellar ataxia Type I represents a mixed group of ataxias (as outline in Table 1), involving presentation of a broad array of neurological symptoms in addition to cerebellar ataxia (Hekman and Gomez, 2015). Additional symptoms include upper limb postural tremor in SCA12 and SCA27, myoclonus and task-specific dystonia in SCA14, sensorineural hearing loss in SCA37 and cognitive impairments in SCA18, SCA13 and SCA21. The most prevalent SCAs (SCA1-3) are caused by trinucleotide repeat expansion within affected genes, leading to polyglutamine (polyQ) tract expansion. The SCAs caused by trinucleotide repeat expansions are typically more severe, with faster progression and multiple sites of neuropathology (Rüb et al., 2013; Matilla-Dueñas et al., 2014). Further, in the trinucleotide repeat SCAs, age of onset and disease severity are correlated with the length of the repeat expansion (Paulson, 2018).

Autosomal dominant cerebellar ataxia Type II is a highly specialized ataxia, whereby classical cerebellar ataxia is accompanied by retinal degeneration (Hekman and Gomez, 2015). Currently, SCA7 is the only spinocerebellar ataxia clinically categorized in this specialized sub-group. SCA7 patients experience progressive blindness due to dystrophy of retinal rods and cones (David et al., 1997; Benton et al., 1998).

Autosomal dominant cerebellar ataxia Type III, also known as pure cerebellar ataxias, are disease states in which the cerebellar ataxia is the sole or predominant manifestation of neurological disease (Hekman and Gomez, 2015). Examples of Type III SCAs are outlined in Table 1. Whilst patients may initially present with pure cerebellar pathology, other neurological symptoms such as extrapyramidal symptoms or cognitive deficits may develop as the disease progresses. For example, SCA6 is caused by a polyglutamine trinucleotide repeat expansion within the causative gene, similar to SCA1, SCA2 and SCA3, but it is considered as a pure cerebellar ataxia as its pathology is predominately confined to the cerebellum. Further, SCA6 is unique compared to other polyQ SCAs in that a shorter repeat length (21-33 repeats) has been found to cause disease (Zhuchenko et al., 1997; Matsuyama et al., 1999; Toru et al., 2000).

## What Pathophysiological Changes Within the Cerebellum Underlie SCA Diseases?

The neuropathology of SCA diseases is varied and complex; in some SCAs, pathology is restricted to the cerebellum whilst other SCAs yield more extensive neuropathology, affecting efferent regions including the thalamus, red nucleus and spinal cord (Paulson et al., 2017). Nevertheless, degeneration and ultimate loss of cerebellar neurons is a neuropathological hallmark of the SCA diseases. Once most spinocerebellar patients display motor symptoms, irreversible damage has already occurred within the brain (Matilla-Dueñas et al., 2014). As the disease progresses heterotopy, altered spatial organization of cells, and atrophy can be detected within the cerebellum by neuroimaging and histological analysis (Matilla-Dueñas et al., 2014).

### Dysfunction and Degeneration of Purkinje Cells

The cerebellum is the second largest brain area, second to the cerebral cortex, but it contains more neurons than the rest of the brain combined (Prestori et al., 2019). Since the early 20th century, the cerebellum has been implicated in the control and timing of movement. Functionally, the cerebellum is critical to ensuring movements are performed precisely, in a coordinated and timely manner (Chopra and Shakkottai, 2014). More recently, the cerebellum has also been appreciated for its role in higher order cognitive and emotional processes (Schmahmann and Caplan, 2006).

In order to achieve smooth and balanced muscle movement the cerebellum receives input from the sensory systems, the spinal cord, and other parts of the brain. Firstly, the cerebellum receives input from the inferior olive in the form of climbing fibers and the spinocerebellar tract in the form of mossy fibers (Jörntell, 2017). These inputs project, both directly and indirectly, to the deep cerebellar nuclei (DCN) present within the white matter of the cerebellum.

Superficial to the DCN are three laminar structures called the granular, Purkinje and molecular layers. The innermost layer, the thick granular layer, is densely packed with granule cells. Superficial to this, is the Purkinje layer, a narrow zone that contains the cell bodies of Purkinje cells, relatively large inhibitory neurons that act to modify activation of the DCN (Huang and Verbeek, 2019). The outermost layer called the molecular layer contains the expansive dendritic trees of Purkinje cells and the parallel fibers of the granule cells intercepting these dendrites at right angles. This layer also contains two types of interneurons called stellate cells and basket cells. Together the excitatory parallel fibers and inhibitory (γ-aminobutyric acid, GABAergic) stellate and basket cells regulate firing of Purkinje cells. From there, the Purkinje cells are the sole neuronal output from the cerebellar cortex, sending GABAergic inputs to the DCN and in turn projecting to the thalamus, brainstem and motor cortex (Huang and Verbeek, 2019).

Dysfunction of Purkinje cells and cerebellar circuitry is thought to be the predominant overlapping pathogenic mechanism across SCA diseases, eliciting hallmark ataxic symptoms (Kasumu and Bezprozvanny, 2012). In fact, Purkinje cells are the most affected neuronal population in the Spinocerebellar ataxias, with some SCAs resulting in loss of more than 75% of the total Purkinje cell population (Kasumu and Bezprozvanny, 2012). The cause of this neurodegeneration of cerebellar Purkinje cells is not well understood but may be due to increased susceptibility to genetic or functional insults than other neuronal cell types (Hekman and Gomez, 2015). Healthy Purkinje cells display autonomous pacemaker activity, firing in absence of synaptic input and with very little variability between spiking intervals (Heiney et al., 2014; Meera et al., 2016; Paulson et al., 2017; Huang and Verbeek, 2019).

**FIGURE 1:**
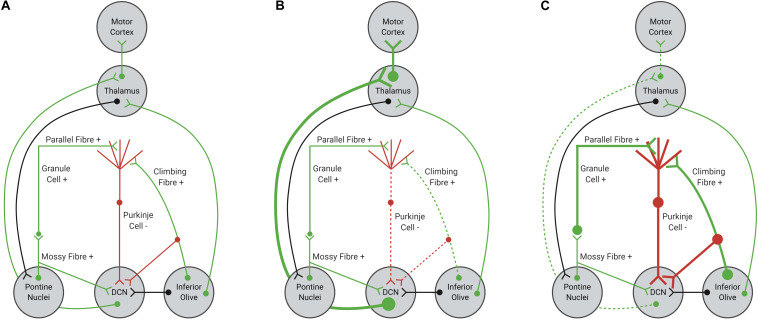
Schematic representation of cerebellar afferents and efferents. **(A)** Normal function of Purkinje cells results in balanced excitation and inhibition in cerebellar circuitry. **(B)** Reduced excitatory input from glutamatergic climbing fibers, due to shortened length or impaired functionality, can decrease Purkinje cell firing, resulting in disinhibition of the DCN-thalamus-motor cortex circuit. **(C)** Increased excitatory input from parallel and climbing fibers, due to increased synaptic connections or increased glutamate signaling within the Purkinje cell synapse, can increase Purkinje cell firing, resulting in inhibition of the DCN-thalamus-motor cortex circuit. Excitatory inputs are highlighted in green, with inhibitory inputs highlighted in red. Black inputs can be either inhibitory or excitatory.

Purkinje cells provide mostly inhibitory inputs to neurons within the DCN, which are critical for planning, performing and fine-tuning motor actions (Heiney et al., 2014; Lee et al., 2015; Huang and Verbeek, 2019; Figure 1A). If firing of Purkinje cells were impaired or dysfunctional, DCN neurons could become disinhibited, increasing excitatory tone within the cerebellum (Meera et al., 2016; Figure 1B). Increased excitation of DCN neurons would result in increased excitatory input from the DCN to motor centers, causing impairments to motor performance, specifically coordination and fine-tuning of movement (Kasumu and Bezprozvanny, 2012; Meera et al., 2016). Indeed, optogenetic silencing of Purkinje cell output in mice evokes increased bursting within DCN neurons and rapid body movements (Heiney et al., 2014; Lee et al., 2015). In contrast, increased excitation of Purkinje cells would result in an overall increase in inhibitory tone, decreasing neurotransmission in the DCN and output to motor centers (Figure 1C). Hence, Purkinje cells are a critical relay center within cerebellar circuits and any disruption to normal Purkinje cell function would alter the function of the cerebellum overall, leading to cerebellar ataxia (Heiney et al., 2014; Lee et al., 2015; Meera et al., 2016; Huang and Verbeek, 2019).

### The Role of Transcriptional Dysregulation in Purkinje Cell Dysfunction

Interestingly, disease-causing proteins linked with polyQ repeat disorders are consistently associated with transcriptional regulation, despite sharing little sequence homology (aside from the polyQ stretch) or functionality (Gerber et al., 1994; Helmlinger et al., 2006). Further, some of the disease-causing proteins are themselves transcription factors, such as expanded ataxin-7 in the case of SCA7 and TATA-binding protein (TBP) in SCA17 (Helmlinger et al., 2004; Shah et al., 2009). Transcriptomic analysis of these SCA genes has revealed that many causative genes (21 of the 23 analyzed) share conserved transcription factor binding sites, suggesting a convergent role of transcription factors involved in the regulation of SCA genes (Bettencourt et al., 2014). In their study, Bettencourt et al. (2014) conducted co-expression network analysis of 687 SCA patient brain samples and identified *ITPR1* to be enriched within SCA transcripts, highlighting altered calcium homeostasis as an overlapping pathogenic mechanism across SCAs. This led to a hypothesis that polyQ disease proteins yield toxic effects through dysregulation of transcription (Gerber et al., 1994; Butler and Bates, 2006; Matilla-Dueñas et al., 2014). Furthermore, it has been suggested that polyQ expansion can inhibit the function of histone acetyltransferases, decreasing histone acetylation and thus decreasing transcriptional activity (Jung and Bonini, 2007; Chou et al., 2014). More recently, altered Purkinje cell transcripts have been identified as a potential pathogenic mechanism for the SCAs, with multiple transcriptional changes reported to affect the function of signaling cascades essential to Purkinje cell function.

Indeed, ATXN1 has been shown to interact with transcriptional regulators and suppress the function of genes such as retinoid and thyroid hormone receptors (SMRT), nuclear receptor co-expressor 1 (NCoR), growth factors (GFI-1) and polyglutamine binding protein 1 (PQBP1) (Butler and Bates, 2006; Lam et al., 2006). The pathogenesis of SCA3 has also been associated with transcriptional dysregulation, as the ataxin-3 protein is hypothesized to act as a histone binding protein, interacting and binding with transcriptional regulators such as CREB-response binding protein (CBP), TBP, histone deacetylase (HDAC) 3, HDAC6 and NCoR (Evert et al., 2006). PolyQ-expansion within the ataxin-3 protein is thought to increase the extent of histone binding, affecting histone acetylation (Evert et al., 2006). Furthermore, it has also been suggested that mutated polyQ proteins can also inhibit the function of histone acetyltransferase (Minamiyama et al., 2004; Jung and Bonini, 2007; Chou et al., 2014). In contrast to the findings of Evert et al. (2006), polyQ-expanded ataxin-3 was found to impair histone acetyltransferase activity in SCA3 mice, resulting in histone hypoacetylation (Chou et al., 2014). Transgenic mice expressing ataxin-3 with 79 polyglutamine repeats also exhibited downregulated cerebellar expression of IP3R1, vesicular glutamate transporter type 2 (VGLUT2) and TBP-interacting protein (Chou et al., 2008). Functionally, the described transcriptional downregulation was found to alter the function or Purkinje cells in *ex vivo* cerebellar slices from ataxin-3-79Q mice.

Ataxin-7, the protein encoded by *ATXN7*, has been identified to form transcription coactivator complexes and regulate gene expression (Helmlinger et al., 2004). PolyQ-expanded ataxin-7 has been found to colocalize with nuclear transcription factors such as CBP, TATA-binding protein-associated factor 4 (TAF4) and specificity protein 1 (SP1), forming neuronal intranuclear inclusions (Helmlinger et al., 2006). Transcriptional dysregulation of ion channel genes has been identified in the cerebellum of SCA7 mice (Chou et al., 2010; Stoyas et al., 2020), suggesting this pathogenic mechanism may be shared across different SCAs or polyQ diseases.

### Transcription Factors or Repressors Implicated in Purkinje Cell Dysfunction

#### Capicua

The toxicity of the ataxin- 1 protein in SCA1 mouse models has been attributed to the formation of large stable complexes with the mammalian homolog of Drosophila Capicua (CIC), a transcriptional repressor, within cerebellar Purkinje cell nuclei (Lam et al., 2006). Furthermore, this toxicity was found to be polyQ-dependent, as expression of mutant S776A polyQ-expanded ATXN1, a non-pathogenic form of the disease-associated protein, resulted in a distinct lack of CIC complexes and neuronal dysfunction in both cellular and *Drosophila* models (Lam et al., 2006). Interestingly, knockout of CIC in SCA1 mice caused improvements in motor performance (Fryer et al., 2011). Whilst this finding may suggest that polyQ expansion of ATXN1 causes a reduction in CIC function, the authors hypothesized that mutant ATXN1 may cause CIC to bind more tightly to transcriptional targets, causing simultaneous hyper-repression and de-repression. Rousseaux et al. (2018) further characterized the role of the ATXN1-CIC complex in SCA1 cerebellar pathology, finding that the ATXN1-CIC complex confers a toxic gain-of-function effect in transgenic SCA1 mice, driving reduced transcription of critical genes in Purkinje cells.

More recently, Chopra et al. (2020) expanded on the findings of Rousseaux et al. (2018), highlighting regional differences in Purkinje cell degeneration and correlating these changes with regional patterns of transcriptional dysregulation. Interestingly, several ion channel genes, such as *KCNMA1*, *CACNA1G*, *TRPC3*, *ITPR1*, and *GRM1*, were found to be downregulated in anterior Purkinje cells isolated from SCA1 mice (Chopra et al., 2020). Furthermore, this neuronal population was also found to highly express CIC (Chopra et al., 2020). Collectively, the findings from Chopra et al. (2020) provide the first experimental evidence associating altered Purkinje cell physiology and impaired calcium homeostasis with transcriptional dysregulation in SCA1, a consequence of altered ATXN1-CIC interactions.

#### RORα

Retinoid-related orphan receptor α (RORα) is a transcription factor that is enriched in cerebellar Purkinje cells and functionally involved in normal Purkinje cell development (Chen et al., 2013). RORα is critical for the retraction of transient Purkinje cell dendrites, facilitating development of the mature dendritic tree (Chen et al., 2013). Deletion of RORα from adult Purkinje cells resulted in regression of immature characteristics such as innervation from multiple functional climbing fibers and invasion of parallel fiber synaptic space (Chen et al., 2013). One of the first animal models of human ataxia, the staggerer mutant mouse, was later found to lack functional RORα (Hamilton et al., 1996). AAV-mediated knock down of RORα also lead to degeneration of Purkinje cell layer alignment, dendritic atrophy and ataxia (Yasui et al., 2020), suggesting that loss of RORα function may be involved in SCA disease phenotypes.

Examination of transgenic mouse models that express pathogenic forms of ATXN1 highlighted the critical role of RORα in cerebellar development. SCA1 transgenic mice display reduced expression of RORα and reduced expression of genes regulated by RORα (Serra et al., 2006). Furthermore, SCA1 transgenic mice share phenotypic overlap with staggerer mice (Crespo-Barreto et al., 2010), suggesting loss of RORα function in SCA1. Interestingly, the phenotypic similarities between SCA1 mice and staggerer mice are not shared with transgenic SCA7 mice, suggesting the transcriptional changes are not caused from polyQ expansion or cerebellar dysfunction, but more likely due to a specific interaction between ATXN1 and RORα (Crespo-Barreto et al., 2010). In their study, Serra et al. (2004) found that four genes regulated by RORα, *ITPR1*, *SLC1A6*, *PCP4(L7)* and *PCP4*, were found to be downregulated in the cerebella of both SCA1-82Q mice and stagger mice. Interestingly, mice expressing a non-pathogenic form of ataxin-1 containing 82 glutamine repeats were found to express similar levels of these genes and RORα to wild type mice (Serra et al., 2006), suggesting loss of RORα function can be attributed to the pathogenicity of expanded, mutant ataxin-1. Delayed expression of mutant ataxin-1 until maturation attenuated Purkinje cell susceptibility to neurodegeneration, turning off expression of ATXN1 resulted in a restoration of RORα expression (Serra et al., 2006). Hence, ATXN1 interacts with RORα to supress expression of RORα-regulated genes that are critical to Purkinje cell development.

In line with findings from staggerer mice and SCA1 mice, transgenic models of SCA3 also display reduced expression of RORα (Konno et al., 2014). Whilst the ataxin-1 protein is found to directly interact with RORα and forming protein complexes (Serra et al., 2006), the ataxin-3 protein does not directly bind with RORα (Watanave et al., 2019), suggesting divergent underlying pathogenic mechanisms.

#### PGC1α and Sirtuin 1

Peroxisome proliferator activated receptor γ coactivator-1α (PGC-1α) is a transcriptional coactivator functionally involved in mitochondrial biogenesis (Lin et al., 2005; Wareski et al., 2009). Deficits in expression or activation of PGC-1α have been associated with motor impairments and neurodegeneration (Lucas et al., 2012). PGC-1α is highly expressed within the cerebellum and PGC-1α knockout mice develop ataxia and movement deficits (Lucas et al., 2015). At the molecular level, knockout of PGC-1α results in a 30% loss of Purkinje cells and downregulation of genes involved in synaptic, structural and metabolic functions (Lucas et al., 2015). PGC-1α was implicated in the regulation of parvalbumin, a calcium binding protein, highlighting a more complex role within the neuronal circuitry (Lucas et al., 2015).

More recently, transcription factor binding-site analysis highlighted downregulation of genes targeted by sirtuin 1 (Sirt1) in SCA7 transgenic mice (Stoyas et al., 2020). Sirt1, an NAD^+^-dependent deacetylase, that has previously been associated with increased survival and reduced age-related neurodegeneration, was found to rescue transcriptional abnormalities in SCA7 mice (Stoyas et al., 2020). One of the identified transcriptional abnormalities was acetylation of PGC-1α. Overexpression of Sirt1 was found to rescue transcriptional abnormalities and motor deficits in SCA7 mice (Stoyas et al., 2020). These recent findings further highlight the role of PGC-1α in healthy Purkinje cell function and metabolism.

### What Makes Cerebellar Purkinje Cells so Sensitive to Changes in Neuronal Signaling?

The spinocerebellar ataxias have been associated with a broad suite of pathological changes to Purkinje cells, including altered cellular morphology, connectivity, physiology, cell number and the appearance of toxic protein species (Chopra and Shakkottai, 2014). Indeed, mutations found in SCA1, 6, 15/16, 19/22, 27, 42 have been found to produce irregular or diminished Purkinje cell signaling (Hoxha et al., 2018). This is contrasted with SCA2, 3, 5, 41 and 44, which have been found to enhance Purkinje cell excitability and increase signaling (Hoxha et al., 2018). However, it is hypothesized that the context of disease proteins may be important for toxicity, as disease proteins may alter the function of protein interactors, resulting in concomitant loss and gain-of-function effects. These concomitant effects are particularly evident in SCA1 and SCA3 (Fogel et al., 2015; Watson et al., 2017). Collectively, these findings paradoxically indicate that both increased and decreased function of disease affected proteins can cause similar deficits to Purkinje cell signaling, detrimentally impacting cerebellar output and movement (Meera et al., 2016). However, it remains unclear why genetic insults lead to Purkinje cell degeneration and spinocerebellar ataxia. Here we outline three distinct properties associated with Purkinje cell function that may increase vulnerability to the pathological consequences of SCA-causing genetic mutations.

#### High Metabolic Activity and Expansive Dendritic Arbor

In order to understand how and why Purkinje cells are so susceptible to the insults caused by in the genetic mutations underlying SCAs, it is interesting to compare Purkinje cells with other cell types within the cerebellum. Morphologically, Purkinje cells are one of the largest neuronal cell types within the cerebellum and exhibit high metabolic activity (Hekman and Gomez, 2015). Purkinje cells consume a substantial amount of ATP, due to the continuous, pacemaker firing properties (Fukumitsu et al., 2016). Purkinje cells also develop expansive dendritic arbors, a property which distinguishes them from other neuronal types within the cerebellum. Recent evidence has suggested that Purkinje cell dendritic development may be highly reliant on regulated mitochondrial fission and transport (Fukumitsu et al., 2016). Disruption of mitochondrial fission in post-mitotic Purkinje cells can lead to enlarged and impaired distribution of mitochondria throughout dendrites, reducing the energy supply to distal dendrites (Fukumitsu et al., 2016). In conditions of reduced energy supply, Purkinje cell dendrites are significantly shorter and less branched, suggesting appropriate energy supply may be required for normal development of the Purkinje cell arborization (Almajan et al., 2012; Fukumitsu et al., 2016). In alignment with this hypothesis, Stoyas et al. (2020) found metabolic dysregulation and depletion of nicotinamide adenine dinucleotide (NAD^+^) in the SCA7 mouse cerebellum. NAD^+^ depletion has also been observed in the nucleus of neurons derived from SCA7 patient induced pluripotent stem cells (Ward et al., 2019). Interestingly, increasing the availability of cellular energy, via treatment with nicotinamide riboside, ameliorated motor deficits and Purkinje cell morphological abnormalities in SCA7 transgenic mice (Stoyas et al., 2020). Furthermore, treatment with creatinine yielded significant therapeutic benefit in a transgenic mouse model of SCA3, improving motor performance and reducing neuronal degeneration (Duarte-Silva et al., 2018). Hence, it is plausible that the high metabolic activity, large cell size and expansive dendritic arbor of Purkinje cells may leave them more vulnerable to changes in the cellular environment (Hekman and Gomez, 2015).

#### Synaptic Plasticity and Glutamate-Induced Excitotoxicity

Structurally, Purkinje cells have substantial dendritic arbors which that receive large volumes of excitatory input (Hekman and Gomez, 2015). Climbing fiber-Purkinje cell and parallel fiber-Purkinje cell synapses undergo activity-dependent plasticity, evoking changes in Purkinje cell physiology (Bushart et al., 2016; Jayabal et al., 2016; Smeets and Verbeek, 2016; Huang and Verbeek, 2019). Increased glutamatergic input can trigger a surplus of climbing fiber-Purkinje cell synapses, with dysfunction of the protein kinase C pathway, shown to increase the number of climbing fiber-Purkinje cell synapses (Shuvaev et al., 2011; Prestori et al., 2019). In contrast, decreased glutamatergic input can reduce connectivity. Histological analysis of ataxin-1 mice revealed a reduced number of climbing fiber-Purkinje cell synapses in the distal segment of the Purkinje cell dendritic arbor (Barnes et al., 2011). Shortened climbing fiber length, resulting in failure to synapse with Purkinje fiber dendrites has also been attributed to reduced Purkinje cell signaling in the pathogenesis of SCA1 (Ebner et al., 2013). It is plausible that changes to climbing fiber connectivity could be a disease mechanism shared across forms of SCA.

Moreover, long-term depression (LTD) at the parallel fiber-Purkinje cell synapse has traditionally been associated with motor learning. Precise control of Ca^2+^ signaling is required for LTD to occur at the parallel fiber-Purkinje cell synapse (Llano et al., 1994). Indeed, impaired calcium signaling has been associated with impaired LTD induction at the parallel fiber-Purkinje cell synapse in ataxin-3 79Q transgenic mice (Chou et al., 2008). Collectively, these phenotypes highlight decreased inhibitory action of Purkinje cells, due to insufficient excitatory input from synapsing climbing fibers.

#### Dysfunction of Purkinje Cell Physiology

Channels located within neuronal synapses are critical to maintaining neuronal excitability; reduced expression or functionality of channels could contribute to altered Purkinje cell excitability. There are three main types of channels; synaptic channels, which include glutamate or GABA receptors, voltage-gated ion channels which that generate ionic currents in response to membrane potential changes and ligand-gated ion channels, which are activated by secondary messengers such as neurotransmitters (Shakkottai and Paulson, 2009). Mutation of channels has been attributed to a range of neurological diseases including forms of ataxia, epilepsy and myotonic disorders, known collectively as channelopathies (Shakkottai and Paulson, 2009). In addition, changes to channel availability and localization, despite a lack of channel mutation, can also lead to alterations in intracellular trafficking and the regulation of membrane potential within the cerebellum, producing ataxia (Shakkottai and Paulson, 2009). Disease-associated mutations can cause toxic gain-of-function effects, dominant-negative effects and haploinsufficiency of channel function, with all three forms of mutation described in SCAs (Shakkottai and Paulson, 2009). There is evidence to suggest that changes to expression of channels and receptors which regulate membrane excitability may precede motor dysfunction and in cell death (Hourez et al., 2011; Kasumu et al., 2012a; Jayabal et al., 2015). Therefore, it is of interest to increase understanding of the basic mechanisms underlying impaired cerebellar channel function so that treatments can be developed to target these mechanisms before cerebellar circuits incur irreversible damage.

Afferent stimulation of Purkinje cells, via climbing fiber or parallel fiber synapses, is mediated by glutamatergic activation of ionotropic AMPA receptors and metabotropic glutamate receptors (mGluRs) located within the synaptic membrane, resulting in release of glutamate (Meera et al., 2016). Furthermore, activation of mGluRs can increase intracellular calcium signaling via activation of the protein kinase C (PKC) pathway, which increases calcium concentrations through cation channels, such as the transient receptor potential type 3 (TRPC3), and the inositol 1,4,5- trisphosphate receptor (IP3R1)(Berridge and Irvine, 1984; Llano et al., 1994; Taylor and Traynor, 1995; Venkatachalam et al., 2003).

Impaired regulation of calcium signaling within cerebellar Purkinje cells is one of the most characterized disease mechanisms underlying spinocerebellar ataxias. Genetic mutation of voltage-gated calcium channels has been identified in both SCA6 and SCA42, however mutations in downstream calcium signaling molecules, such as IP3R, are also prolific within SCA subtypes. Furthermore, mutation of voltage-gated potassium or sodium channels can also contribute to altered Purkinje cell excitability. Whilst AMPA receptor function remains relatively spared in SCAs, loss of mGluR function, or downstream glutamate signaling, has been identified in SCA5 and SCA44. Ultimately, each of these disease mechanisms alter synaptic neurotransmission, leading to progressive cerebellar dysfunction and pervasive loss of Purkinje cells resulting in SCA motor phenotypes (Huang and Verbeek, 2019).

##### Homeostatic control of calcium signaling

Homeostatic control of intracellular calcium release is a functionally important cellular mechanism, as calcium ions (Ca^2+^) can act as an intracellular messenger initiating other processes such as synaptic neurotransmission and transcriptional regulation (Berridge and Irvine, 1984; Taylor and Traynor, 1995). Furthermore, appropriate calcium concentrations are required to facilitate long term potentiation (LTP) and long term depression (LTD) within the cerebellum (Llano et al., 1994). Excessive intracellular calcium can induce toxicity and detrimentally impact on neuronal cell survival (Kasumu and Bezprozvanny, 2012).

**FIGURE 2:**
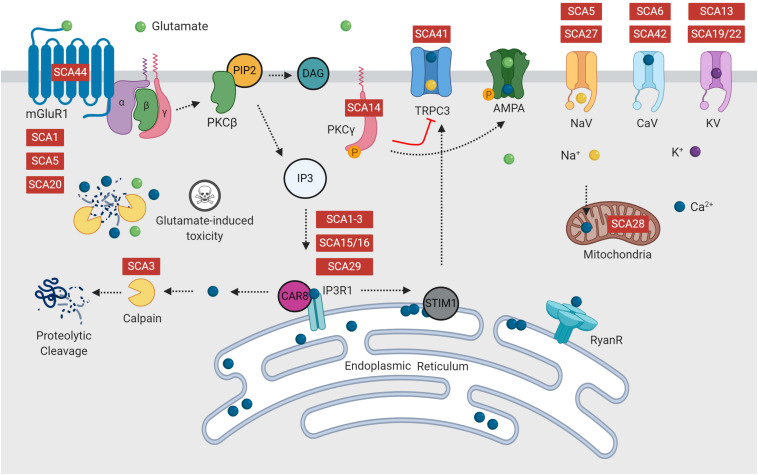
Many forms of spinocerebellar ataxia are attributed to mutations in intracellular calcium signaling, glutamate release or the PKC pathway, which form multiple positive feedback loops. Disruption of calcium and glutamate signaling is hypothesized to initiate Purkinje cell dysfunction, resulting in ataxia and eventual cell death. Diseases indicated in red are reported to have dysfunction of this component of the signaling pathway.

Increased Ca^2+^ influx triggers activation of the PKC signaling pathway (Berridge and Irvine, 1984). Activation of mGluRs via binding with glutamate, activates a signaling cascade, ending with release of Ca^2+^ from the endoplasmic reticulum via activation of IP3R1 (Figure 2). This initial release of Ca^2+^ into the cytosol can be further amplified via activation of ryanodine receptors (RyanR), an intracellular Ca^2+^ release channel (Berridge and Irvine, 1984; Llano et al., 1994; Taylor and Traynor, 1995). Depletion of endoplasmic reticulum calcium stores triggers translocation of stromal interaction molecule 1 (STIM1), an endoplasmic reticulum calcium sensor, to the peripheral endoplasmic reticulum cisternae, triggering activation of TRPC3 channels (Prestori et al., 2019). It is well regarded that disrupted calcium signaling may initiate cellular processes, which eventuate in cerebellar Purkinje cell death (Kasumu and Bezprozvanny, 2012; Matilla-Dueñas et al., 2014; Bushart et al., 2016; Hisatsune et al., 2018; Prestori et al., 2019).

##### Mutation of voltage-gated calcium channels in SCA diseases

Voltage-gated calcium channels regulate the influx of Ca^2+^ into neurons during membrane depolarization. As such, voltage-gated calcium channels modulate neuronal excitability and can trigger calcium-dependent processes including release of neurotransmitters, synaptic plasticity and gene transcription (Cain and Snutch, 2011). High-voltage P/Q-type (Cav2.1), N-type (Cav2.2) and R-type (Cav2.3) channels are considered as the primary driver of evoked synaptic transmission (Cain and Snutch, 2011). Functionally, Cav2.1 channels are critical to the regulation of spiking properties and contribute to calcium spikes triggered by increased activity of cerebellar climbing fibers. In contrast, T-type voltage-gated calcium channels (Cav3.1, Cav3.2 and Cav3.3) are activated by lower voltage thresholds and exhibit a hyperpolarised range of activation and inactivation, making them ideally suited to regulate neuronal activity (Zamponi et al., 2015; Hashiguchi et al., 2019). T-type channels are typically inactivated, however postsynaptic inhibitory events can recover T-type channels from inactivation, facilitating “rebound bursting” (Zamponi et al., 2015; Hashiguchi et al., 2019). Voltage-gated calcium channels have also been found to functionally interact with large conductance calcium-activated potassium (BK) channels, providing additional modulation of neuronal excitability through BK channel activity (Zamponi et al., 2015).

The presence of a mutation in the *CACNA1A* gene, which encodes the α1A-subunit of voltage-gated P/Q-type calcium channels (Cav2.1), results in an array of neurological disorders including SCA6, episodic ataxia type 2 and familial hemiplegic migraine type 1 (Zhuchenko et al., 1997; Matsuyama et al., 1999; Toru et al., 2000). Each of the neurological disorders is associated with a different *CACNA1A* mutation, suggesting differential effects on Ca^2+^ signaling (Zhuchenko et al., 1997). Initially, SCA6 pathogenesis was thought to stem from dysfunction of the voltage gated calcium channel Cav2.1, which is encoded by *CACNA1A* gene (Zhuchenko et al., 1997; Matsuyama et al., 1999; Toru et al., 2000). However, experimental animal models expressing mutant *CACNA1A* failed to consistently yield deficits in Cav2.1 function (Matsuyama et al., 1999; Toru et al., 2000). More recently, *CACNA1A* was identified as a biscistronic gene, encoding both the α1A subunit of the P/Q type voltage-gated calcium channel Cav2.1 and α1ACT, a transcription factor which shares sequence homology with the cytoplasmic c-terminal of the α1A subunit (Matsuyama et al., 1999; Toru et al., 2000). Interestingly, the polyQ expansion is present within both independently translated proteins. Functionally, α1ACT is involved in cerebellar development and enhances expression of genes essential for Purkinje cell survival, such as GRN, PMCA2 and BTG1 (Du et al., 2013, 2019). PolyQ-expanded α1ACT exhibits reduced transcription factor function and produces nuclear toxicity, resulting in ataxia and neuronal death, driving SCA6 pathology (Du et al., 2013, 2019; Bavassano et al., 2017; Pastor et al., 2018).

Recently, genetic sequencing lead to the identification of a single point mutation in the *CACNA1G* gene in ten SCA42 families (Coutelier et al., 2015; Morino et al., 2015; Kimura et al., 2017; Ngo et al., 2018; Hashiguchi et al., 2019). This mutation, which causes an arginine to histidine change, alters the voltage-sensing region of T-type voltage-gated calcium channel Cav3.1 (Hashiguchi et al., 2019). Functionally, this mutation is thought to impair Purkinje cell “rebound bursting,” requiring higher voltage thresholds to elicit depolarization of Purkinje cells (Hashiguchi et al., 2019). To further confirm if this point mutation is the phenotypic driver of SCA42 pathology, a transgenic mouse model (Cacna1g-Arg1723His) has been generated (Hashiguchi et al., 2019). Cacna1g-Arg1723His mice recapitulated many pathological hallmarks of human SCA42, including early adulthood onset of ataxia and cerebellar atrophy (Hashiguchi et al., 2019). Interestingly, Cacna1g-Arg1723His mice did not display any direct impairments to Purkinje cell signaling. Nevertheless, the firing of inferior olivary neurons was found to be altered, enhancing activity of climbing fiber-Purkinje cell synapse and thus Purkinje cell excitability (Hashiguchi et al., 2019). Interestingly, Cacna1g-Arg1723His mice share phenotypic similarities with Cav3.1 knockout mice (Matsumoto-Makidono et al., 2016), suggesting loss of Cav3.1 function in SCA42 (Hashiguchi et al., 2019).

##### Dysfunction of calcium signaling within the protein kinase C pathway

Spinocerebellar ataxia type 14 is caused by mutation to of the *PRKCG* gene (Brkanac et al., 2002; Morita et al., 2006), which encodes the PKCγ isoform of the PKC subfamily (PKCγ) and is activated by binding with the secondary messenger DAG, in the presence of phosphatidylserine (Berridge and Irvine, 1984; Saito and Shirai, 2002). Currently, over 40 different mutations in *PRKCG* have been attributed to SCA14, with some mutations causing a loss of PKCγ function, whilst other mutations enhance PKCγ function (Verbeek et al., 2005; Adachi et al., 2008; Shuvaev et al., 2011; Ji et al., 2014). Increased intracellular levels of Ca^2+^ stimulate PKCγ to translocate from the cytosol to the plasma membrane (Berridge and Irvine, 1984; Venkatachalam et al., 2003; Prestori et al., 2019). PKCγ is expressed throughout the central nervous system and is specifically localized to neurons (Saito and Shirai, 2002). In the cerebellum, PKCγ is highly expressed within Purkinje cells and is implicated in the normal development of climbing fiber input from the inferior olive (Chen et al., 1995; Kano et al., 1995; Shuvaev et al., 2011). It is likely that PKCγ may be critical to normal Purkinje cell function and hence may be an important therapeutic target. Several SCA14 mutations have been found to enhance the intrinsic activity of PKCγ (Adachi et al., 2008), leading to decreased binding with DAG and decreased inhibition of extracellular Ca^2+^ entry through TRPC3 receptors (Venkatachalam et al., 2003). Absence of PKCγ-mediated control of TRPC3 channel activity causes prolonged, aberrant increases in intracellular Ca^2+^ signaling in Purkinje cells (Venkatachalam et al., 2003). Transgenic mice expressing an S361G mutation in PKCγ display severe loss of PKCγ function, evidenced by inhibited dendrite development, Purkinje cell degeneration and ataxia (Ji et al., 2014), aligning with cerebellar deficits found in human SCA14 patients.

Interestingly, there is considerable experimental evidence to suggest abnormal IP3R1 function, a downstream signaling protein found within the PKCγ pathway, in the pathogenesis of many spinocerebellar ataxias and other neurodegenerative diseases including Huntington’s disease and Alzheimer’s disease (Tada et al., 2016; Prestori et al., 2019). IP3Rs are intracellular inositol triphosphate-3 (IP3) channels which that release stored calcium from the endoplasmic reticulum (Berridge and Irvine, 1984; Llano et al., 1994; Taylor and Traynor, 1995). IP3Rs, in addition to other intracellular calcium channels such as RyanRs, are critical to maintaining free calcium concentrations within the cytoplasm (Berridge and Irvine, 1984; Llano et al., 1994; Taylor and Traynor, 1995). IP3Rs are specifically expressed in the central nervous system, with high expression in cerebellar Purkinje cells, the basal ganglia and thalamus (Llano et al., 1994; Tada et al., 2016). Furthermore, IP3R1 is the key receptor type responsible for regulating climbing fiber and parallel fiber inputs to Purkinje cell dendrites, linking synaptic signaling with LTP and LTD (Llano et al., 1994; Huang and Verbeek, 2019). IP3R1, via binding with IP3, mediates the spaciotemporal activity of calcium oscillations which are required for the induction of LTD (Llano et al., 1994; Huang and Verbeek, 2019).

Homozygous *ITPR1* knockout mice, which are deficient in cytosolic IP3-mediated Ca^2+^ release, display diminished LTD, severe ataxia and tonic-clonic seizures, resulting in death by weaning age (Matsumoto et al., 1996). Mutation of the *ITPR1* gene, which encodes IP3R1, results in both SCA15/16 and SCA29 (Storey et al., 2001; van de Leemput et al., 2007). Most causes of SCA15 are attributed to haploinsufficiency of IP3R1 and suppressed calcium signaling, however one mutation (P1059L) has been found to increase IP3 affinity with no effect on calcium signaling (Ando et al., 2018). In contrast, eleven missense mutations in *ITPR1* have been associated with SCA29, however the functional consequences of the mutations are relatively unknown (Ando et al., 2018). Most SCA29 mutations induce dominant-negative effects, abolishing IP3R1 activity or carbonic anhydrase-related protein VIII (CAR8)-mediated regulation of IP3R1, resulting in decreased calcium release, whilst the R36C mutation induces a toxic gain-of-function, enhancing calcium release (Ando et al., 2018).

In calcium imaging and electrophysiology experiments, Chen et al. (2008) evidenced increased sensitivity of IP3R1 channels and potentiation of IP3R-mediated calcium release in MJD/SCA3 patient fibroblasts compared with healthy controls. This study went further to show preferential binding between full length IP3R1 and expanded ataxin-3 proteins (77Q and 127Q), but not wild type ataxin-3 in both cells and mice modeling SCA3. This finding was also replicated *in vivo*, with anti-ataxin 3 monoclonal antibodies precipitating IP3R1 from cortical lysates obtained from SCA3-YAC-84Q transgenic mice, but not wild type controls (Chen et al., 2008). Collectively, these findings suggest that mutant ataxin-3 interacts with IP3R1, resulting in elevated calcium signaling. Similarly, Liu et al. (2009) found that pathological forms of the ataxin-2 protein, but not wild type ataxin-2, also interact with the cytosolic C-terminal region of IP3R1s, consequently increasing IP3R1 activation and IP3R1-mediated Ca^2+^ responses in cultured Purkinje cells (Liu et al., 2009). Furthermore, investigation of IP3R1-mediated Ca^2+^ signals in cultured Purkinje cells revealed that inhibition of RyanR1 could attenuate calcium signals and protect against glutamate-induced toxicity (Liu et al., 2009). *In vivo* experimentation also revealed amelioration of disease phenotypes in ataxin-3 and ataxin-2 transgenic mice following chronic treatment with dantrolene, a clinically approved compound used for the treatment of hyperthermia and muscle spasticity (Chen et al., 2008; Liu et al., 2009). Due to the similarities in binding, it has been hypothesized that the polyQ tract within expanded ataxin-2 and ataxin-3 specifically binds to IP3R1, producing toxic enhancement of IP3R1 function (Chen et al., 2008; Liu et al., 2009). Treatments that target or modulate IP3R1s may be beneficial to multiple SCAs.

Many proteins involved in maintaining calcium homeostasis have been found to be downregulated in animal models of SCA1, including IP3R1, CAR8, Cav3.1, TRPC3 and sarco-endoplasmic reticulum Ca^2+^ ATPase 2 (Lin et al., 2000; Serra et al., 2004; Prestori et al., 2019). Furthermore, SCA1 transgenic mice also displayed reduced expression of calcium binding proteins, including calbindin and parvalbumin (Vig et al., 1998). It is hypothesized that downregulation of calbindin and parvalbumin may impair the ability of Purkinje cells to appropriately buffer excessive calcium, which is further exacerbated by reductions in calcium binding proteins (Bushart et al., 2016). Accordingly, suppression of calbindin in SCA1 mice enhanced the severity of SCA1 phenotypes, worsening motor performance and neuronal loss (Vig et al., 2012). In contrast, knockout of ASIC1a, acid-sensing channel isoform 1a, which transmits inward calcium currents upon activation, improved motor performance and increased intensity of calbindin and parvalbumin staining (Vig et al., 2014). Collectively, the findings of Vig and colleagues suggest that in SCA1 transgenic mice, intracellular calcium handling may be impaired due to reduced expression of the calbindin protein, consequently increasing the sensitivity of Purkinje cells to altered calcium homeostasis. Similar findings were also reported in SCA7-92Q mice, with immunohistochemical examination revealing reduced expression of Cav3.1 and calbindin in the posterior cerebellum (Stoyas et al., 2020). This finding is of particular interest as altered calcium homeostasis had not previously been associated with SCA7, providing further evidence of disrupted calcium homeostasis as a central pathogenic mechanism across SCAs.

#### Dysfunction of Other Ionotropic Channels

SCA5 and SCA27 share similar pathophysiology, with both diseases resulting in reduced Purkinje cell excitability due to reduced expression or activation of voltage-gated sodium channels. Reduced excitability of Purkinje cells could result in decreased signaling at the Purkinje cell-DCN synapse, reducing excitation of DCN projections to motor centers (Chopra and Shakkottai, 2014). Mice deficient in beta-III spectrin, encoded by the gene *SPTBN2*, mutated in SCA5 patients (Ikeda et al., 2006), displayed decreased Purkinje cell firing and worsening with disease progression (Perkins et al., 2010). Electrophysiological slice recordings from beta-III spectrin knockout mice demonstrated reduced spontaneous firing rates, reduced total sodium current and reduced resurgent sodium current (Perkins et al., 2010). Mutation of beta-III spectrin has been found to affect localization of membrane bound protein in dendrites and cell bodies, exerting a dominant negative effect on sodium channel complexes.

The pathogenesis of SCA27 has been attributed to mutation of fibroblast growth factor 14 (*FGF14*), which binds with voltage-gated sodium channels and overall modulating neuronal excitability (Yan et al., 2013). *FGF14* knock out mice showed failed spontaneous firing, degraded responsiveness to depolarizing current, loss of Nav1.6 within Purkinje cells and ataxia, aligning with the disease features present in SCA27 patients (Laezza et al., 2007). Furthermore, cultured neurons expressing mutated *FGF14* displayed decreased sodium channel currents and neuronal excitability, suggesting a dominant-negative effect (Laezza et al., 2007; Shakkottai et al., 2009). It is hypothesized that mutant FGF14 may disrupt the function of wild-type FGF14 protein which acts to stabilize sodium channel expression, thus leading to altered expression of voltage-gated sodium channels (Laezza et al., 2007; Shakkottai et al., 2009). Furthermore, FGF14 was also found to regulate voltage-gated calcium channels, Cav2.1 and Cav2.2 (Yan et al., 2013). Expression of pathological FGF14 in cerebellar granule cells exerted a dominant-negative effect on Cav2.1 and Cav2.2, reducing Ca^2+^ influx and diminishing excitatory postsynaptic currents at the granule cell-Purkinje cell synapse (Yan et al., 2013). Evidence suggests that FGF14 can regulate multiple ionic currents, therefore the pathogenesis of SCA27 may be mediated by decreased function of both voltage-gated sodium channel and voltage-gated calcium channels, leading to reduced output of Purkinje cells (Yan et al., 2013).

Spinocerebellar ataxia type 13 (SCA13) is caused by loss of Kv3.3 function (Waters et al., 2006; Figueroa et al., 2010). Kv3.3 is functionally involved in depolarizing both somatic sodium spikes and dendritic calcium spikes within Purkinje cells, with some expression in granule cells and DCN neurons. Therefore, loss of voltage-gated potassium channel function leads to neuronal hyperexcitability and impaired calcium homeostasis (Irie et al., 2014; Bushart et al., 2016). Expression of mutant *KCNC3* (R424H) resulted in disrupted dendritic development, altered Purkinje cell spiking and cell death in cerebellar cultures (Irie et al., 2014). Interestingly, SCA13 mutations are capable of both decreasing and increasing Kv3.3 activity (Waters et al., 2006; Figueroa et al., 2010). Furthermore, exome sequencing of two SCA19/22 families lead to the identification of a missense mutation in *KCND3*, which encodes the voltage-gated potassium channel Kv4.3 (Duarri et al., 2012; Lee et al., 2012). Mutation of *KCND3* causes a toxic loss-of-function, as Kv4.3 is retained within the endoplasmic reticulum instead of localizing to the plasma membrane, resulting in LTP impairments at the Purkinje cell synapse (Duarri et al., 2012; Lee et al., 2012).

Emerging evidence suggests that dysfunction of calcium-activated potassium channels can be attributed to increased intrinsic excitability of Purkinje cells in SCA1, SCA2 and SCA7. In ataxin-1 82Q-expressing mice, expression of large-conductance calcium-activated potassium (BK) channels and G-protein coupled inwardly rectifying potassium (GIRK1) channels is decreased with disease progression. Furthermore, Dell’orco et al. (2015) observed a correlation between decreased Purkinje cell firing and increased conduction of K^+^. Virally mediated overexpression of BK channels improved motor performance and rescued dendritic degeneration in SCA1 mice (Dell’orco et al., 2015). More recently, Stoyas et al. (2020) observed irregular spiking patterns and reduced capacitance in Purkinje cells of the posterior cerebellum in transgenic mice expressing ataxin-7 with a polyglutamine length of 92. The authors hypothesized that this neurophysiological abnormality was due to dysfunction of calcium activated potassium channels, and indeed found reduced expression of BK channels within the cerebellum (Stoyas et al., 2020). Furthermore, viral overexpression of BK transcripts improved the regularity of Purkinje cell spiking Stoyas et al. (2020), highlighting similarities between SCA1 and SCA7.

The pathogenesis of SCA1 may also be attributed to altered potassium channel function, leading to imbalance in the depolarizing and hyperpolarizing currents and consequently altered neuronal spiking (Bushart et al., 2016). Hence, impairments in Purkinje cell firing in SCA1 may be underscored by impaired BK channel function. Similarly, SCA2 mice expressing ataxin-2-128Q within Purkinje cells failed to maintain neuron spiking due to a reduction in transcript levels of *KCNMA1* (Dell’orco et al., 2017). Therefore, dysfunction of voltage-gated potassium channel may contribute to irregular Purkinje cell spiking in animal models of SCA.

#### Glutamate Signaling Within the Cerebellum

Glutamate is the most abundant neurotransmitter within the mammalian brain. Under normal conditions, glutamate can trigger transient increases in calcium levels within Purkinje cells via activation of mGluRs and ionotropic, such as AMPA receptors and NMDA receptors (Piochon et al., 2010; Kasumu et al., 2012a; Watson et al., 2017). Historically, Purkinje cells were thought to lack NMDA receptors, relying on other glutamatergic receptors to modulate glutamate signaling (Perkel et al., 1990; Llano et al., 1991). However, the presence of NMDA receptors was confirmed at the climbing fiber-Purkinje cell synapse (Piochon et al., 2007, 2010), suggesting a role of NMDA receptors within cerebellar circuitry.

Binding of glutamate to mGluRs at the Purkinje cell synapse activates a complex cascade, the PKC pathway, which increases intracellular release of calcium. In contrast, activation of AMPA or NMDA receptors triggers membrane depolarization, activating voltage-gated calcium channels and cytoplasmic Ca^2+^ influx (Kasumu and Bezprozvanny, 2012). Activation of mGluR, AMPA or NMDA receptor evokes a transient increase in cytosolic calcium levels, eliciting excitation of Purkinje cells (Kasumu and Bezprozvanny, 2012).

Metabotropic glutamate receptor type 1 (mGluR1), encoded by *GRM1* gene, is one of the most highly expressed receptor types within the mammalian central nervous system, with particularly high abundance in Purkinje cells (Watson et al., 2017). Neuronal excitability is modulated by mGluRs, which yield slow excitatory post-synaptic currents, and excitatory amino acid transporters (EAATs), which transport glutamate (Power and Empson, 2014). Glutamate re-uptake from the Purkinje cell presynaptic cleft is critical for precise signaling and is modulated by EAAT1 (GLAST), EAAT2 (GLT1) and EAAT4 (Power and Empson, 2014; Bushart et al., 2016). Moreover, EAAT4 modulates mGluR1-mediated events at the parallel and climbing fiber synapses, influencing plasticity (Power and Empson, 2014). A reduction in the expression of EAATs or mGluRs would result in prolonged excitatory synaptic transmission, as glutamate is not being effectively removed from the synapse (Power and Empson, 2014). Furthermore, increased activation of Purkinje cells would in turn, increase inhibitory inputs received by the DCN, resulting in defective cerebellar output (Power and Empson, 2014). Deletion of GRM1 has been shown to cause an ataxic phenotype (Conquet et al., 1994), whilst restoration of normal mGluR1 expressing in Purkinje cells can rescue motor impairments (Ichise et al., 2000; Nakao et al., 2007). This evidence highlights the critical role of mGluR1 signaling in Purkinje cell development and function.

#### Evidence for Disrupted Glutamate Signaling in SCAs

Recently, missense mutation of *GRM1* was identified in three SCA44 families (Watson et al., 2017). Expression of mutant mGluR1 was found to dramatically enhance mGluR1 activity, evoking a toxic gain-of-function and producing excitotoxicity within Purkinje cells via a positive-feedback loop (Watson et al., 2017). Increased excitatory input triggers increased release of glutamate and thus mGluR1 activation, which in turn produces increased in intracellular calcium levels. However, increased intracellular calcium levels act to potentiate mGluR1-mediated signals, thus promoting increased downstream calcium signaling (Watson et al., 2017). Whilst mutation of *GRM1* is relatively rare, dysfunction of mGluR1 and other downstream components of the mGluR1signalling pathway have previously been associated with spinocerebellar ataxias. The deleterious impacts of reduced mGluR1 function are highlighted in transgenic knockout models, which evidence functional deficits such as altered LTD, abnormal Purkinje cell innervation and ataxia (Aiba et al., 1994; Ichise et al., 2000; Rossi et al., 2013).

Impairments in mGluR1 function has also been observed in animal models of human spinocerebellar ataxia, including SCA1, SCA2 and SCA3 (Notartomaso et al., 2013; Prestori et al., 2019). In SCA3, mGluR1 is mislocalized, resulting in reduced synaptic expression and disruption of normal mGluR1 signaling (Konno et al., 2014). In contrast, SCA1 and SCA2 has been associated with mGluR1 hyperactivity, a consequence of prolonged elevations in Ca^2+^ concentrations (Power et al., 2016; Meera et al., 2017). It is likely that increases in glutamate load and reduced calcium buffering capacity within Purkinje cells may combine to form a deleterious positive feedback loop, further propagating mGluR and calcium signaling disturbances, consequently driving Purkinje cell dysfunction (Meera et al., 2016, 2017). Recent evidence has highlighted that changes to mGluR signaling and reduced amplitude of mGluR-mediated excitatory post-synaptic potentials may precede degeneration of Purkinje cell dendrites (Paulson et al., 2017). Impairment of mGluR-mediated signaling may also impair LTD of Purkinje cell-parallel fiber synapses, resulting in altered motor learning and motor deficits (Paulson et al., 2017; Huang and Verbeek, 2019)

Transient receptor potential type 3 channels are functionally involved in mGluR1-mediated slow post-synaptic currents and found within the same protein complex as mGluR1, localizing to Purkinje cell soma and dendrites (Becker, 2014; Prestori et al., 2019). Furthermore, TRPC3 channels functionally interact with mGluR1s to induce LTD and mediate extracellular Ca^2+^ entry (Venkatachalam et al., 2003; Becker et al., 2009). Activity of TRPC3 is negatively regulated by phosphorylation of PKCγ; loss of PKCγ-mediated inhibition may enhance glutamate and calcium signaling (Venkatachalam et al., 2003; Becker, 2014). Transgenic mice expressing a missense mutation in *TRPC3*, moonwalker mice, display enhanced TRPC3 function resulting in ataxic gait and Purkinje cell loss (Becker et al., 2009). Recently, a point mutation in *TRPC3* was implicated in an adult-onset form of ataxia (Fogel et al., 2015), now classified as SCA41. This mutation was found to infer a toxic gain-of-function, increasing Purkinje cell degeneration and sharing phenotypic similarities with moonwalker mice (Becker, 2017). It was hypothesized that this toxic gain-of-function mutation causes calcium overload, resulting in Purkinje cell dysfunction and degeneration (Becker, 2014).

As previously described, SCA5 has been found to be caused by mutation of the *SPTBN2* gene, which encodes the beta-III spectrin protein (Ikeda et al., 2006). Beta-III spectrin is functionally involved in stabilizing EAAT4 at the Purkinje cell membrane (Ikeda et al., 2006; Perkins et al., 2010). More recently, mutation of beta-III spectrin was also found to interact with the α-subunit of mGluR1 (mGluR1α) causing mislocalization of mGluR1α in Purkinje cell dendritic spines, decreased responsiveness of postsynaptic mGluR1 and impaired mGluR1-mediated LTP (Armbrust et al., 2014). Therefore, loss of beta-II spectrin function in SCA5 likely results in excessive glutamate presence within the synapse and glutamate toxicity in the postsynaptic neuron (Bushart et al., 2016). Furthermore, reduced EAAT-4 expression has also been observed in the cerebellum of transgenic SCA1 mice (Lin et al., 2000; Serra et al., 2004).

Despite different underlying pathogenic mechanisms initiating neuronal dysfunction and degeneration, increased exposure to glutamate has been shown accentuate toxicity. Application of the mGluR1/5 agonist, DHPG, resulted in increased amplitude of calcium transients in cultured Purkinje cells expressing pathogenic ataxin-2 and ataxin-3 (Chen et al., 2008; Liu et al., 2009). Furthermore, direct application of glutamate was found to increase neuronal death and increase production of toxic protein fragments in cellular models of SCA2 and SCA3 (Liu et al., 2009; Koch et al., 2011). Therefore, increased glutamate exposure may act to further enhance the toxic effect of other disease mechanisms in models of SCA.

### What Makes Cerebellar Purkinje Cells so Sensitive to Changes in Calcium and Glutamate Signaling?

Under normal conditions, transient increases in intracellular calcium levels are well tolerated, however more prolonged increases can be damaging (Kasumu and Bezprozvanny, 2012). Purkinje cells maintain calcium homeostasis via expression of calcium channels, calcium sensors, calcium buffers and calcium-sensitive kinases (Llano et al., 1994; Kasumu and Bezprozvanny, 2012). It is hypothesized that as Purkinje cells progressively degenerate, their ability to regulate intracellular calcium homeostasis is reduced (Meera et al., 2016). There are two specific mechanisms by which Purkinje cells manage supraphysiological calcium levels; calcium binding proteins and mitochondrial up-take of excess calcium ions (Llano et al., 1994). There is considerable evidence to suggest one or both mechanisms may be disrupted in transgenic animal models and human SCA patients (Kasumu and Bezprozvanny, 2012; Vig et al., 2012; Bushart et al., 2016; König et al., 2016; Prestori et al., 2019).

Firstly, excess intracellular Ca^2+^ can be taken up calcium binding proteins calbindin and parvalbumin (Llano et al., 1994; Bastianelli, 2003). These calcium binding proteins are found exclusively within GABAergic interneurons, with calbindin expressed within Purkinje cells, whilst parvalbumin is expressed within basket cells, stellate cells and Golgi cells (Bastianelli, 2003). Calcium binding proteins are enriched within Purkinje cells and act as the first line of defense for calcium buffering (Llano et al., 1994; Bastianelli, 2003; Kasumu and Bezprozvanny, 2012). It is hypothesized that the relatively high expression levels of calcium binding proteins within Purkinje cells may accommodate large Ca^2+^ influxes that occur as a result of repetitive neuronal spiking and the increased metabolic load of Purkinje cells (Bushart et al., 2016). The important neuroprotective role of endogenous calcium binding proteins are highlighted in transgenic calbindin knockout mice, which present with mild ataxic symptoms and impaired Purkinje cell physiology (Airaksinen et al., 1997; Bastianelli, 2003; Kasumu and Bezprozvanny, 2012; Bushart et al., 2016). Furthermore, knockout of both calbindin and parvalbumin in ataxin-1-expressing mice resulted in an exacerbated the phenotype, producing severe ataxia and altered Purkinje cell morphology (Vig et al., 2012).

As a second line of defense, some excess Ca^2+^ can be stored by Purkinje cell mitochondria (Kasumu and Bezprozvanny, 2012). However, mitochondria can quickly become overloaded, initiating activation of calcium sensitive enzymes including phosphatases and kinases, potentially altering gene transcription and proteases such as calpains, which can cleave and degrade cellular substrates (Kasumu and Bezprozvanny, 2012). Increased activation of nitric oxide synthase in turn increases production of nitric oxide, which can trigger DNA damage and mitochondrial dysfunction (Kasumu and Bezprozvanny, 2012). Interestingly, mutation of *AFG3L2*, which encodes the m-AAA subunit of the mitochondrial metalloprotease AFG3L2 (ATPase family gene 3-like 2) has been found to underlie SCA28 (Di Bella et al., 2010; Almajan et al., 2012; König et al., 2016). Functionally, this mutation causes a dominant negative effect on of m-AAA protease activity (Di Bella et al., 2010), resulting in accumulation of mitochondrial Ca^2+^ uniporter expression (König et al., 2016). Post mortem analysis of SCA28 patient brain tissue has revealed mitochondrial swelling within Purkinje cells (Di Bella et al., 2010; Almajan et al., 2012).

**FIGURE 3:**
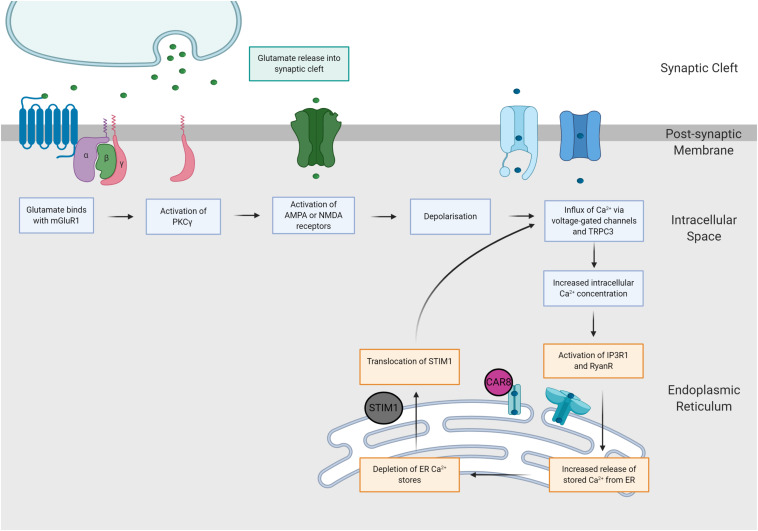
The mGluR-Ca^2+^ hypothesis of SCA pathogenesis. Increases in glutamatergic signaling consequently increase intracellular calcium concentrations via two distinct pathways. Firstly, glutamate binds with AMPA and NMDA receptors, eliciting neuronal depolarization and influx of calcium. Secondly, glutamate binds with mGluR1 receptors, causing activation of the PKCγ pathway, which increases intracellular calcium concentrations via activation of IP3R1 and TRPC3.

It is possible that when Purkinje cells are exposed to prolonged supraphysiological levels of intracellular calcium, SCA phenotypes may be caused by one convergent SCA pathology, regardless of the underlying genetic mutation (Meera et al., 2017). This hypothesis, the mGluR-Ca^2+^ of SCA pathogenesis, suggests that the ultimate degeneration and loss of Purkinje cells is mediated by two potent positive feedback mechanisms (Meera et al., 2016; Figure 3). Firstly, Batchelor and Garthwaite (1997) demonstrated that increased intracellular Ca^2+^ results in potentiation of mGluR-mediated signals. Hence, elevated levels of Ca^2+^ within Purkinje cells activates a positive feedback loop, producing further increases in intracellular Ca^2+^ via increased TRPC3 currents and IP3R1-mediated release (Llano et al., 1994; Taylor and Traynor, 1995; Meera et al., 2016). In addition, the indirect interaction between IP3R1 activity and Ca^2+^ is governed by a bell-shaped curve, whereby both low and high concentrations of intracellular calcium elicit potentiation of IP3R1-mediated Ca^2+^ release, whilst moderate intracellular Ca^2+^ concentrations inhibit IP3R1 activity (Bezprozvanny et al., 1991; Finch et al., 1991; Llano et al., 1994). Experimental evidence gathered from SCA2 transgenic mice provides support for this hypothesis, demonstrating SCA2 phenotypes are exacerbated by positive feedback mechanism, linking elevations in basal calcium concentrations with increased mGluR1 signaling and IPR31-mediated release of intracellular calcium (Meera et al., 2017). As IP3R1 is uniquely expressed within Purkinje cells, it is plausible that these distinct positive feedback loops may increase the vulnerability of Purkinje cells to calcium overload, ultimately enhancing influx of calcium to the intracellular space (Llano et al., 1994; Meera et al., 2016). Furthermore, this mechanism of rising calcium overload could potentially explain the varied progression of different forms of SCA (Meera et al., 2016). For example, SCA1, 2 and 3 are widely regarded to pertain impaired mGluR1 or IP3R1 function (Lin et al., 2000; Chen et al., 2008; Liu et al., 2009) and are additionally, considered to be more severe forms of disease (Paulson et al., 2017). Further insight into calcium overload and the mGluR-Ca^2+^ excitotoxicity hypothesis of SCA may reveal a positive correlation between disease severity and dysfunction of mGluR and/or IP3R1.

## Treatments Aimed at Rectifying Impaired Cerebellar Physiology

Currently, treatment of the SCAs is predominately aimed at alleviating disease symptoms. Evidence gathered from transgenic mouse models of SCA suggest that pathogenic changes in Purkinje cell neurophysiology are correlated with the onset of motor deficits and early stages of neuronal loss (Bushart et al., 2016). Therefore, it is hypothesized that electrophysiological dysfunction may be an early contributor to disease pathogenesis and that treatments which target these early disease states may aid to slow the progression of motor dysfunction and neurodegeneration (Bushart et al., 2016). Therapeutic approaches that aim to correct impaired modulation of ion release or improve glutamate re-uptake may improve synaptic physiology in SCAs (Bushart et al., 2016).

### Treatments Aimed at Rectifying Transcriptional Dysregulation

Transcriptional regulation is mediated by two enzymes, histone deacetylase (HDAC) and histone acetyltransferase (HAT). HDACs act to remove acetyl groups from ε-N-acetyl lysine, causing chromatin remodeling and repression of transcription (Butler and Bates, 2006; Haggarty and Tsai, 2011). In contrast, HAT adds acetyl groups opening chromatin architecture and increasing transcription (Butler and Bates, 2006). Many SCA-associated genes have been found to confer haploinsufficiency or loss-of-function effects, these include *ATXN7* (SCA7), *ITPR1* (SCA15/16, SCA29), *KCND3* (SCA 19/22), *FGF14* (SCA27), *AFG3L2* (SCA28) and *CACNA1G* (SCA42). Considering this, treatments that increase gene transcription may be of therapeutic benefit in the SCAs. One treatment approach is to inhibit the activity HDAC, resulting in increased histone acetylation, chromatin relaxation and increased transcription, ameliorating dysfunction caused by loss-of-function mutations.

In contrast, many SCAs are characterized by gain-of-function gene mutations and it is hypothesized that mutant, expanded polyQ proteins may result in loss of HAT function and abnormally interaction with HDACs (Butler and Bates, 2006), causing transcriptional dysregulation. Therefore, treatments that increase transcription may yield increased toxicity due to increased expression of pathogenic disease proteins. However, many SCAs that yield toxic enhancements in gene function, also yield concomitant loss of interacting partner function. For example, mutant ATXN1 is generally considered to possess enhanced function, however functionality of the transcriptional co-repressor CIC is decreased when interacting with mutant ATXN1 (Rousseaux et al., 2018), resulting in downregulation of many key ion channel channels that are critical for maintaining Purkinje cell function (Chopra et al., 2020). Hence, it can be argued that SCA1 confers concomitant gain and loss of function effects (Lam et al., 2006). Thus, whilst HDAC inhibition may increase translation of mutant polyQ proteins, this negative effect may be outweighed by the neuroprotective benefit of increasing expression of genes that have previously been shown to be downregulated, leading to impaired function of Purkinje cells. In these SCAs, benefit may be enhanced via treatment with HDAC inhibitors that also aid clearance of toxic protein species (Butler and Bates, 2006). In our laboratory, we have found treatment with HDAC inhibitors to yield protective benefits in models of SCA3, rectifying histone acetylation and aiding the clearance of toxic ataxin-3 species (unpublished).

Previous studies have reported that treatment with HDAC inhibitors can have neuroprotective effects for the treatment of polyglutamine repeat diseases (Ferrante et al., 2003; Minamiyama et al., 2004; Butler and Bates, 2006; Jung and Bonini, 2007; Latouche et al., 2007; Chou et al., 2010, 2011). Sodium butyrate, the sodium salt of the short chain fatty acid butyric acid, is a class I and IIa HDAC inhibitor produced naturally during digestion by gut microbiota (Canani et al., 2011). Sodium butyrate has been found to improve movement phenotypes and inhibit HDAC enzyme activity in models of Huntington’s Disease and spinal and bulbar muscular atrophy (Ferrante et al., 2003; Minamiyama et al., 2004). In a transgenic mouse model of SCA3, chronic treatment with sodium butyrate ameliorated histone hypoacetylation and reduced movement deficits (Chou et al., 2011). Furthermore, sodium butyrate decreased neuronal death in primary neuronal cultures derived from ataxin-7 (100Q) expressing rats (Latouche et al., 2007). Sodium valproate (valproic acid or divalproex sodium), a class I and IIa HDAC inhibitor, has also been found to yield improvements in transcriptional dysregulation and improve proteinopathy in animal models of SCA3 (Yi et al., 2013; Lin et al., 2014; Wang et al., 2018; Wang et al., 2019). Furthermore, a Phase I/II clinical trial of valproic acid in SCA3 patients reported improvements to motor function but also minor adverse effects (Lei et al., 2016). Valproic acid has also been found to be beneficial for patients with episodic ataxia type 2, caused by mutation in the CACNA1A gene (Scoggan et al., 2006).

Activation of sirtuin 1 (SIRT1), a class III NAD^+^-dependent histone deacetylase (Butler and Bates, 2006), was recently found to yield neuroprotective effects in a transgenic mouse model of SCA7 through transactivation of calcium regulatory genes (Stoyas et al., 2020). Treatment with the compound nicotinamide riboside increased NAD^+^ availability, increasing the activity of SIRT1 and transcription of SIRT1-mediated genes, consequently improving calcium homeostasis and regularity of spiking in cerebellar Purkinje cells in transgenic mice expressing ataxin-7 with 92 glutamines (Stoyas et al., 2020).

### Treatments Aimed at Correcting Voltage-Gated Ion Channel Function

It is hypothesized that treatments that restore normal Purkinje cell pacemaker firing and reduce activation of calcium-dependent proteases and cell death processes could slow the progression of neurodegeneration (Bushart et al., 2016). Previously, compounds that have been found to modulate Purkinje cell excitability and reduce hyperexcitability have provided neuroprotective benefits in models of SCA (Bushart et al., 2016).

The intrinsic pacemaker firing of Purkinje cell is vital to motor control and coordination and is regulated with small-conductance calcium-activated potassium (SK) channels. Application of SK2/3 channel activator, NS13001, NS309 and CyPPA, on cerebellar slices of SCA2 (ataxin-2 58Q) mice revealed Purkinje cell firing activity improved to tonic firing (Kasumu et al., 2012a). Additionally, ingestion of NS13001 in SCA2 mice had improved motor function and prevented Purkinje cell degeneration (Kasumu and Bezprozvanny, 2012). Similarly, treatment with chlorzoxazone, an SK channel activator, rectified tonic firing of Purkinje cells in SCA 2 mice (Egorova et al., 2016). This led to the clinical study of the safety and efficacy of treatment with chlorzoxazone and baclofen (GABA_A_ receptor agonist) in SCA1 (Bushart et al., 2018).

The therapeutic efficacy of different SK channel activators has also been demonstrated in a preclinical animal models expressing mutations in voltage-gated calcium channel or polyQ expanded proteins including SCA3 (Lorenzon et al., 1998; Waters et al., 2006; Shakkottai et al., 2011; Gao et al., 2012). In contrast, treatments that inhibit voltage-gated potassium channels, such as aminopyridines, which are approved for the treatment of multiple sclerosis, can offer therapeutic potential through the restoration of Purkinje cell firing rate. Specifically, 4-aminopyridine and 3,4-diaminopyridine, were tested on SCA1 (ataxin-1 82Q) mice and found to correct Purkinje cell firing frequency, prevent Purkinje cell degeneration and increased BDNF levels within the cerebellum (Inoue et al., 2001; Hourez et al., 2011). Similarly, acute treatment of cerebellar slices from SCA6 (84Q) mice with 4-aminopyridine increased the precision of Purkinje cell firing (Jayabal et al., 2016). This finding was further confirmed *in vivo*, chronic 4-aminopyridine treatment yielding motor improvements and increased precision of Purkinje cell firing in SCA6 mice (Jayabal et al., 2016).

### Treatments Aimed at Reducing Calcium Signaling

Evidence gathered from preclinical animal models of SCA have suggested treatment with calcium signaling blockers may also offer therapeutic benefit (Chen et al., 2008; Liu et al., 2009). One approach is treatment with dantrolene, a compound which inhibits RyanR-mediated release of Ca^2+^ from the endoplasmic reticulum (Chen et al., 2008; Liu et al., 2009). Chronic dantrolene treatment yielded improved motor performance and prevented cell death in transgenic SCA3 and SCA2 mice (Chen et al., 2008; Liu et al., 2009). In addition, virally mediated overexpression of the inositol 1,4,5-phosphatase enzyme (5PP), ameliorated abnormal Purkinje cell firing, rescued motor performance and reduced Purkinje cell degeneration in ataxin-2 58Q-expressing mice (Kasumu et al., 2012b). Functionally, 5PP converts active IP3 to inactive IP2, inhibiting IP3R-mediated calcium signaling within Purkinje cells (Kasumu et al., 2012b). Furthermore, treatments which that increase expression of calcium binding proteins, calbindin and parvalbumin, may offer therapeutic benefit by enhancing calcium handling capacity, limiting calcium-mediated toxicity (Bushart et al., 2016).

One hypothesized mechanism of disease for SCA3 is increased activity of calcium-activated proteases, such as calpains, which induce proteolytic cleavage of the ataxin-3 protein, resulting in the formation of toxic ataxin-3 fragments (Koch et al., 2011; Hübener et al., 2013). Calpain cleavage sites have been identified along the ataxin-3 protein, supporting the hypothesis of calpain-mediated ataxin-3 proteolysis (Weber et al., 2017). Overexpressing the endogenous calpain inhibitor, calpastatin, in SCA3 murine models prevent ataxin-3 proteolysis, reduce ataxin-3 positive inclusions and prevent neurodegeneration (Simões et al., 2012). Cellular and animal models of SCA3 have also investigated treatment using calpain inhibitor compounds (ALLN, calpeptin, MDL28170, BDA410) and proven success in reducing ataxin-3 cleavage fragments, ataxin-3 aggregation, improving aberrant locomotion and prevent neurodegeneration (Haacke et al., 2007; Koch et al., 2011; Simões et al., 2014; Watchon et al., 2017). One study went further to demonstrate calpain inhibition can remove ataxin-3 cleavage fragments through the protein quality control pathway, macroautophagy, ameliorating disease phenotypes in a zebrafish model of SCA3 (Watchon et al., 2017).

### Treatments Aimed at Reducing Glutamate-Induced Excitotoxicity

Various studies have explored modulating glutamate levels as a therapeutic option in the SCAs affected by glutamate-induce excitotoxicity. One approach that can be utilized to reduce excessive glutamate release is to administer modulators of mGluR and GABA receptor function, effectively increasing or decreasing excitation or inhibition within the cerebellum.

Treatment with JNJ1625968, a negative-allosteric modulator (NAM) of mGluR1, has been reported found to rescue aberrant motor function in SCA1 mice, whilst reducing the prolonged mGluR1 activity of ataxin-1 82Q in *ex vivo* cerebellar slices expressing Purkinje neurons (Power et al., 2016). In contrast, administration of JNJ1625968 treatment *in vivo* worsened the motor phenotype of ataxin-1 154Q mice (Notartomaso et al., 2013). These contrasting findings may be due to differences in the treatment regime used, as 2.5 mg/kg JNJ1625968 was administered to symptomatic 30-week old mice in the Notartomaso et al. (2013) study, whilst the Power et al. (2016) study treated their SCA1 mice with 0.03 mg/kg JNJ1625968 at 12-weeks of age. Hence, the divergent effects of the treatment may be attributed to the different stages of disease progression that the treatment was applied. Additionally, Ro0711401, a mGluR positive-allosteric modulator, was found to improve motor function and increase Purkinje cell dendritic spine length (Notartomaso et al., 2013). FDA-approved compound, nitazoxanide (mGluR1/5- NAM) has also been shown to modulate mGluR1 function, similar to wild-type levels *in vitro* modeling SCA44 (Watson et al., 2017).

Since certain SCAs have similar phenotypes of glutamate-induced excitotoxicity, riluzole has been extensively trialed in animal models as well as clinical trials. Riluzole is known to prevent glutamate release by inhibition of NMDA receptors and is a currently therapeutic option for amyotrophic lateral sclerosis (Doble, 1996). Whilst murine models of SCA1 and SCA3 did not show improvements in motor behavior, prolonged treatment with riluzole affected ataxin-3 proteinopathy by reducing soluble ataxin-3 and accumulating ataxin-3 in cortical cell bodies (Nag et al., 2013; Schmidt et al., 2016). Despite this, riluzole has been tested on SCA patients for safety and efficacy, providing minimal improvement in ataxic symptoms (Ristori et al., 2010; Romano et al., 2015). More recently troriluzole (BHV-4157), a new prodrug of riluzole, was trialed in SCA1-3, SCA6-8 and SCA10 patients in a 48-week and 96-week open label clinical trial. Patients yielded improved SARA scores, suggesting a lack of disease progression during the treatment period (Beiner et al., 2019; Wirtz et al., 2020). Additionally, memantine, an NMDA receptor antagonist, is another compound that has been trialed as a potential therapeutic in for the SCAs. Treatment of SCA1 (ataxin-1 154Q) knock-in mice treated with memantine proved effective by increasing lifespan, reduced ubiquitin positive neuronal inclusions and prevented Purkinje cell neurodegeneration (Iizuka et al., 2015).

Another therapeutic approach examined for the treatment of SCA1 was increasing inhibitory tone via GABA receptor activation. Shuvaev et al. (2017) found that a single dose of baclofen (GABA_B_ receptor agonist) directly into the cerebellum of SCA1 mice was sufficient to improving motor impairment and enhancing mGluR1 signaling between parallel fibers and Purkinje cells and prevent motor impairment. Furthermore, Chopra et al. (2018) demonstrated that combined treatment with baclofen and virally mediated overexpression of BK channels was more effective at increasing Purkinje cell calcium spiking thresholds and reducing motor impairments in ataxin-1 82Q expressing mice than treatment with baclofen alone. This suggests that perhaps combinatorial approaches which modulate glutamate release and other disease mechanisms may be more effective.

## Concluding Remarks

There is a consensus within the field of spinocerebellar ataxia research that more basic science research is required to fully comprehend early disease mechanisms that occur early within the window of reversibility, before the onset of permanent cerebellar damage (Matilla-Dueñas et al., 2014). Increased understanding of disease mechanisms will facilitate the development of new treatment strategies that target pathological changes which prior to neuronal damage and death (Matilla-Dueñas et al., 2014). Whilst a single, unifying, pathogenic mechanism is yet to be identified, it is widely regarded that changes to transcriptional dysregulation, RNA toxicity, impaired proteostasis and altered neuronal signaling are common pathogenic themes across different types of SCA (Matilla-Dueñas et al., 2014; Hekman and Gomez, 2015).

Despite rapid progress in the field of ataxia research and identification of causative genetic mutations, our understanding of precisely how genetic mutations cause insult to Purkinje cells and render cerebellar circuits defective remains limited (Huang and Verbeek, 2019). Interestingly, the consequences of recurrent pathogenic themes align with altered Purkinje cell function. Indeed, dysfunction of Purkinje cell firing has been identified as an early pathogenic hallmark in many SCAs, including SCA1 and SCA2 (Paulson et al., 2017; Hoxha et al., 2018). Identified causes of perturbed Purkinje cell physiology include altered expression of genes critical to Purkinje cell function or development, altered expression or functionality of channels or receptors such as mGluR1, TRPC3 or IP3R1, and changes to excitatory tone, resulting from increased or decreased input from glutamatergic climbing or parallel fibers. As the sole output neuronal population within the cerebellar cortex, dysfunction of Purkinje cell integration can result in both increased and decreased activation of DCN-thalamus-motor cortex projections. Furthermore, impaired Purkinje cell function is thought to be characteristic of the early disease state, preceding motor impairments and toxicity (Shakkottai and Paulson, 2009; Hoxha et al., 2018).

Current treatment approaches aimed at rectifying Purkinje cell dysfunction include increasing gene expression through HDAC inhibitors, pharmacological manipulation of receptor or channel function, and removal of toxic protein species. Whilst current treatment approaches can alleviate disease symptoms and slow progression in preclinical animal models, clinical efficacy is yet to be determined. Therefore, it is beneficial to enhance understanding of disease mechanisms as compounds that correct aberrant physiology may offer enhanced therapeutic potential (Shakkottai and Paulson, 2009; Chopra and Shakkottai, 2014). Further elucidation of the changes that occur early in the disease process prior to toxicity, therapeutic approaches may be better placed to ultimately slow or halt disease progression (Chopra and Shakkottai, 2014; Matilla-Dueñas et al., 2014; Paulson et al., 2017).

## Author Contributions

AL conceptualized the theme of the review. All authors provided an intellectual contribution to the content and direction of the manuscript, and have approved the final version for publication.

## Conflict of Interest

The authors declare that the research was conducted in the absence of any commercial or financial relationships that could be construed as a potential conflict of interest.

## Abbreviations

BK: large conductance calcium-activated potassium
DCN: deep cerebellar nuclei
GABA: γ-aminobutyric acid
HAT: histone acetyltransferase
HDAC: histone deacetylase
LTD: long-term depression
LTP: long-term potentiation
NAM: negative-allosteric modulator
PAM: positive-allosteric modulator
PKC: protein kinase C
polyQ: polyglutamine
SCA: Spinocerebellar ataxia
SK: small conductance calcium-activated potassium.
